# Genome structure and transcriptional regulation of human coronavirus NL63

**DOI:** 10.1186/1743-422X-1-7

**Published:** 2004-11-17

**Authors:** Krzysztof Pyrc, Maarten F Jebbink, Ben Berkhout, Lia van der Hoek

**Affiliations:** 1 Department of Human Retrovirology, University of Amsterdam, Meibergdreef 15, 1105 AZ, Amsterdam, The Netherlands

## Abstract

**Background:**

Two human coronaviruses are known since the 1960s: HCoV-229E and HCoV-OC43. SARS-CoV was discovered in the early spring of 2003, followed by the identification of HCoV-NL63, the fourth member of the coronaviridae family that infects humans. In this study, we describe the genome structure and the transcription strategy of HCoV-NL63 by experimental analysis of the viral subgenomic mRNAs.

**Results:**

The genome of HCoV-NL63 has the following gene order: 1a-1b-S-ORF3-E-M-N. The GC content of the HCoV-NL63 genome is extremely low (34%) compared to other coronaviruses, and we therefore performed additional analysis of the nucleotide composition. Overall, the RNA genome is very low in C and high in U, and this is also reflected in the codon usage. Inspection of the nucleotide composition along the genome indicates that the C-count increases significantly in the last one-third of the genome at the expense of U and G. We document the production of subgenomic (sg) mRNAs coding for the S, ORF3, E, M and N proteins. We did not detect any additional sg mRNA. Furthermore, we sequenced the 5' end of all sg mRNAs, confirming the presence of an identical leader sequence in each sg mRNA. Northern blot analysis indicated that the expression level among the sg mRNAs differs significantly, with the sg mRNA encoding nucleocapsid (N) being the most abundant.

**Conclusions:**

The presented data give insight into the viral evolution and mutational patterns in coronaviral genome. Furthermore our data show that HCoV-NL63 employs the discontinuous replication strategy with generation of subgenomic mRNAs during the (-) strand synthesis. Because HCoV-NL63 has a low pathogenicity and is able to grow easily in cell culture, this virus can be a powerful tool to study SARS coronavirus pathogenesis.

## Background

Until recently only two human coronaviruses were known – human coronavirus (HCoV) 229E and HCoV-OC43, representatives of the group 1 and 2 coronaviruses, respectively. Both were identified in 1960s and are generally considered as common cold viruses. An outbreak of severe acute respiratory syndrome (SARS) in the spring of 2003 led to the rapid identification of SARS-CoV [1,2], which is considered to be a distinct member of the group 2 coronaviruses [3] or the first member of group 4 coronaviruses [4,5]. We identified earlier this year another human pathogen from this family: HCoV-NL63 [6], a variant that belongs to group 1 together with HCoV-229E and PEDV. These recent findings may be striking, as since the 1960's not a single new HCoV had been described. The genome features of SARS-CoV and its transcription strategy have been described in detail [1,5,7]. Here, we present such an analysis for HCoV-NL63.

HCoV-NL63 is a member of the coronaviridae family that clusters together with arteri-, toro- and roniviruses in the order of the nidovirales. Coronaviruses are enveloped viruses with a positive, single stranded RNA genome of approximately 27 to 32 kb. The 5' two-third of a coronavirus genome encodes a polyprotein that contains all enzymes necessary for RNA replication. The expression of the complete polyprotein requires a -1 ribosomal frameshift during translation that is triggered by a pseudoknot RNA structure [8,9]. The polyprotein undergoes autocatalytic cleavage by the viral papain-like proteinase and a chymotrypsin-like proteinase. The 3' one-third of a coronavirus genome encodes several structural proteins such as spike (S), envelope (E), membrane (M) and nucleocapsid (N) that, among other functions, participate in the budding process and that are incorporated into the virus particle. Some of the group 2 viruses encode a hemagglutinin esterase (HE) [10,11]. Non-structural protein genes are located between the structural genes. These accessory genes vary significantly in number and sequence among coronavirus species. Their precise function is unknown, but several reports indicate that they can modulate viral pathogenicity [12].

Coronavirus replication is a complex, not yet fully understood mechanism [13,14]. The 5' end of the genomic RNA contains the untranslated leader (L) sequence with the Transcription Regulation Sequence (TRS) in the downstream part. The L TRS is very similar to sequences that can be found in front of each open reading frame (body TRSs). The RNA-dependent RNA-polymerase has been proposed to pause after a body TRS of a particular gene is copied during (-) strand synthesis, subsequently switching to the L TRS and thus adding a common L sequence to each sg mRNA [15]. This discontinuous transcription mechanism is based on base-pairing of the nascent (-) strand copy RNA with the (+) strand L TRS. The nested set of (-) strand sg mRNAs are subsequently copied into a set of (+) strand sg mRNA. Other factors besides the sequence similarity between body and L TRS influence the efficiency of transcription. The level of transcription of a particular gene has been reported to be inversely related to the distance of a particular TRS to the 3' end of the genome [16-19].

In this study, we analyzed the genome structure of HCoV-NL63. First, we focus on the unusual nucleotide composition of the RNA genome. We describe in detail the bias in the nucleotide composition and its influence on the codon usage of this virus. We provide a possible mechanistic explanation for a shift in nucleotide bias at two-third of the HCoV-NL63 genome that is based on the RNA replication mechanism. Second, we describe in detail the different sg mRNAs generated during HCoV-NL63 replication and their relative abundance.

## Results

### Nucleotide content of the HCoV-NL63 genome

**Figure 1:**
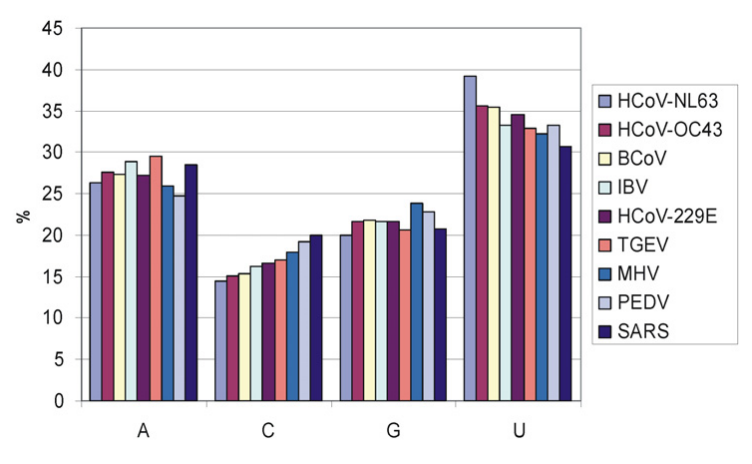
Nucleotide content of coronaviridae RNA genomes. We arranged the viruses based on their C-count, which ranges from 14% (HCoV-NL63) to 20% (SARS-CoV).

We described previously that the newly identified HCoV-NL63 virus has a typical coronavirus genome structure and gene order [6]. The nucleotide composition of the genomic (+) strand RNA of several coronaviridae members is presented in Figure 1, demonstrating a common pattern with U as the most abundant nucleotide and G and in particular C as underrepresented nucleotides. HCoV-NL63 has the most extreme nucleotide bias among the coronaviridae, with 39% U and only 14% C. As a general trend, U and C seem to compete directly, because the genomes with the lowest C-count (HCoV-NL63, HCoV-OC43 and BCoV) have the highest U-count and vice versa (Figure 1).

**Figure 2:**
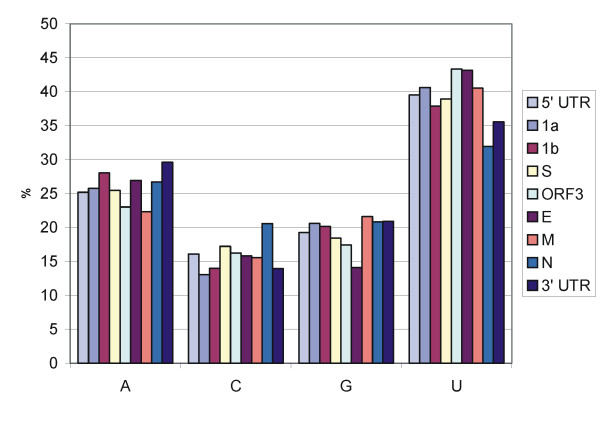
Nucleotide content of individual HCoV-NL63 genes and the 5'/3' untranslated regions (UTR).

To investigate if all coding regions of HCoV-NL63 display a similarly strong preference for U and against C, we also plotted the nucleotide count for the individual genes and 5' and 3' non-coding regions (Figure 2). The typical nucleotide bias is observed in all genome segments. The highest U-count is found in the ORF3 and E genes (43%) and the lowest C-count in the 1a/1b genes and the 3' UTR (13%, 14% and 14%, respectively). The N gene is most moderate in its nucleotide bias, with 21% C and 32% U, confirming the "competition" idea that was already suggested by inspection of Figure 1.

**Figure 3:**
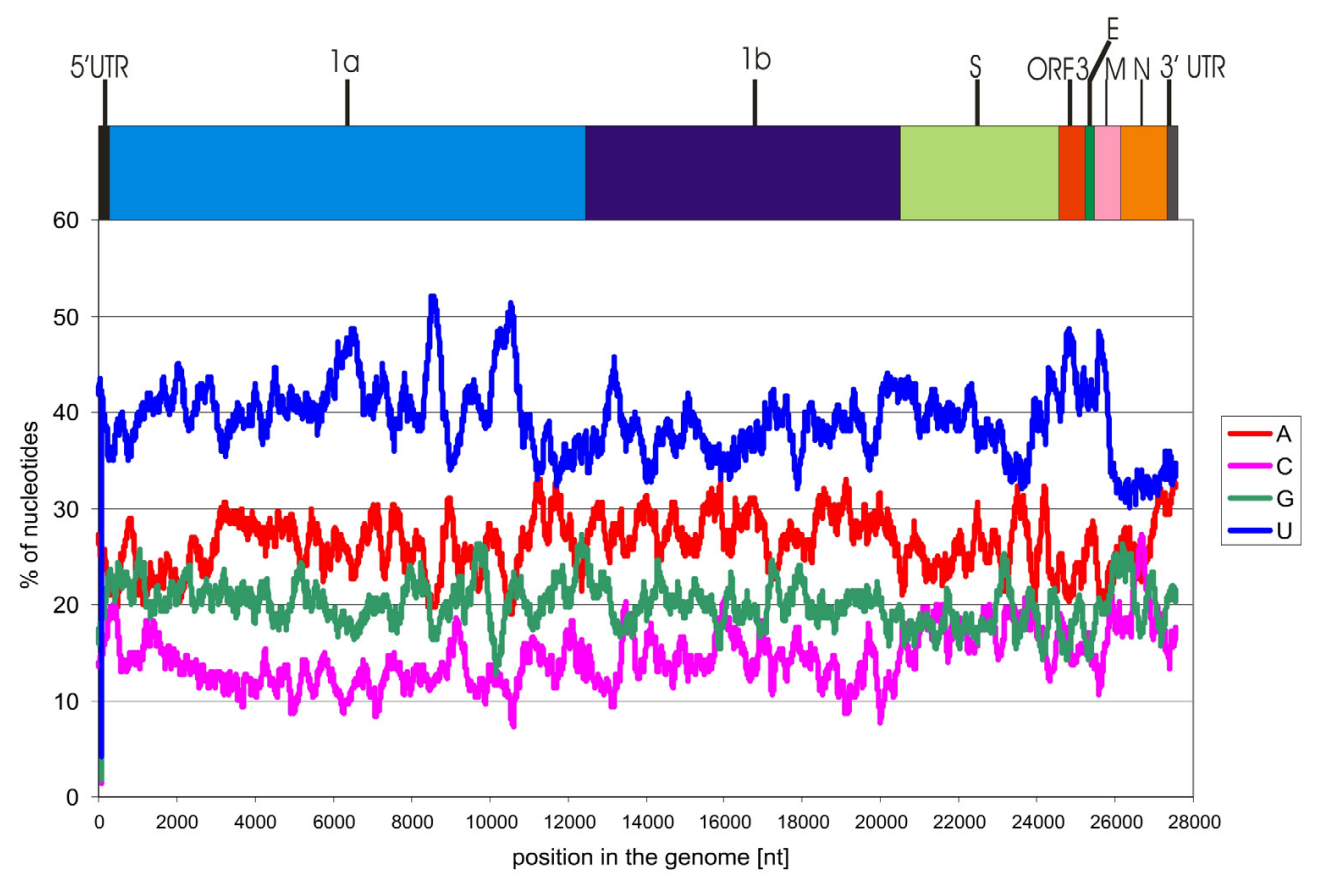
Nucleotide distribution along the HCoV-NL63 genome. The change in the C- and G-count at two-third of the genome is statistically significant for all tested coronaviruses (HCoV-NL63, HCoV-229E, SARS-CoV, HCoV-OC43) with p < 0.01 for C-count and p < 0.05 for G-count in Mann-Whitney U test for two independent samples.

We plotted the nucleotide distribution along the genome (Figure 3) to determine whether there is any significant variation. We observed that local changes in A-count are inversely linked to changes in G-count. This is most striking in the 20400–26000 nt region, where three A peaks are mirrored by three G anti-peaks. Although the typical bias is maintained along the genome, the most notable variation occurs in the last one-third of the genome, where an increase in C and a decrease in G content is apparent. This region encodes the structural proteins.

**Figure 4:**
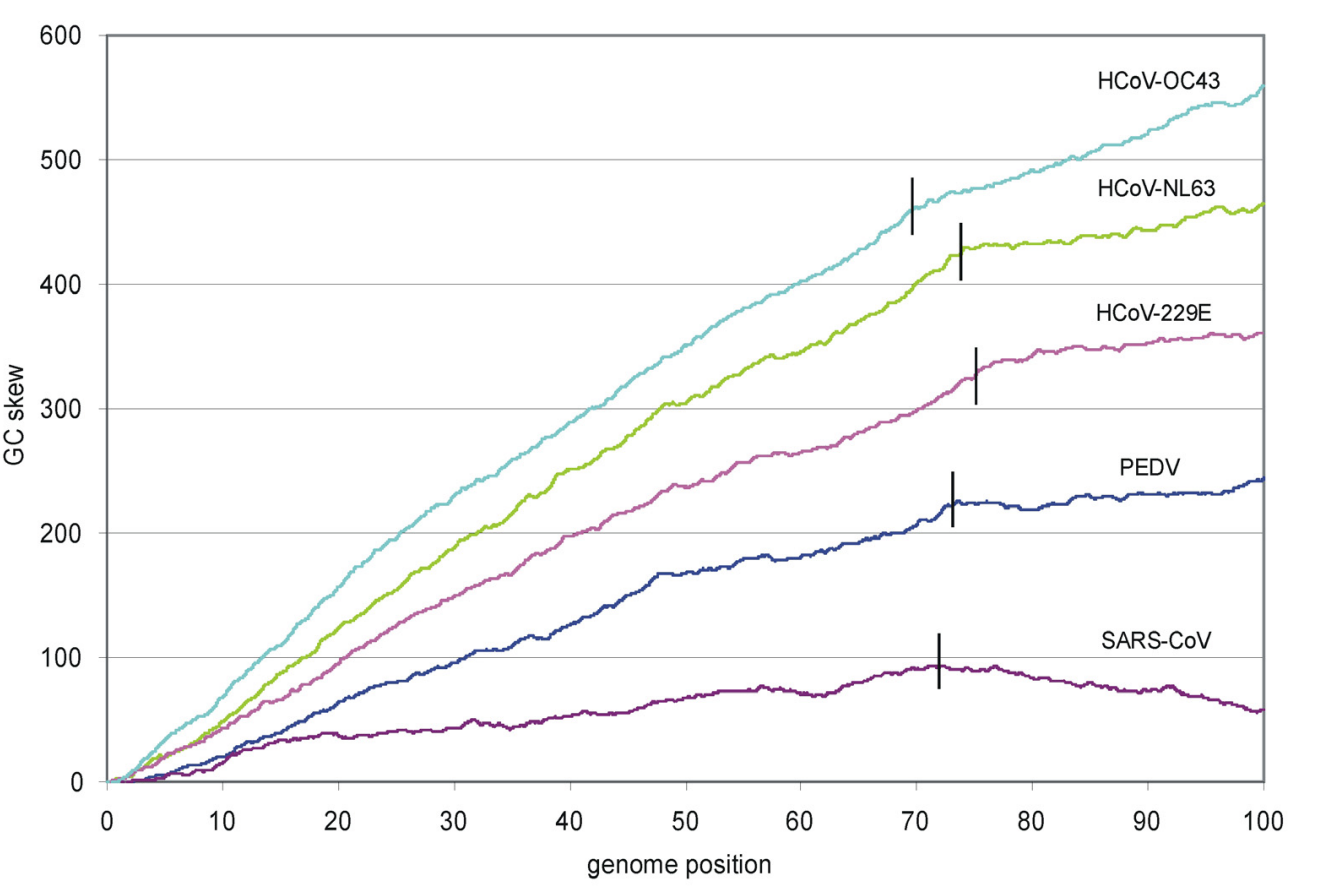
Cumulative GC-skew diagrams for several coronaviral RNA genomes. The vertical bar indicates the border between the 1a/1b and the structural genes.

Recently, Grigoriev reported an interesting feature within coronaviral genomes that is visible when the cumulative GC-skew is plotted [20,21]. Cumulative GC skew graph is a way to visualize the local G:C ratio along the genome, discarding the local fluctuations. A biphasic pattern was described that separates the 1a/1b polyprotein genes and the structural genes. The cumulative GC-skews for HCoV-NL63 and four other coronaviruses: HCoV-OC43, HCoV-229E, PEDV and SARS-CoV are presented in Figure 4. In the 1a/1b genes, the G:C ratio reaches high levels, whereas for all coronaviruses, including HCoV-NL63, the 3' end of the genome displays a flattening of the curve, as the G:C ratio reaches value ~ 1 or less. Grigoriev proposed that this biphasic pattern is due to the discontinuous transcription process [20]. He suggested that the frequent deamination of cytosine on the (-) strand RNA results in a decrease of G on the (+) strand in the region encoding the structural genes. In the discussion section we will present an alternative mechanistic explanation.

### HCoV-NL63 codon usage

**Figure 5:**
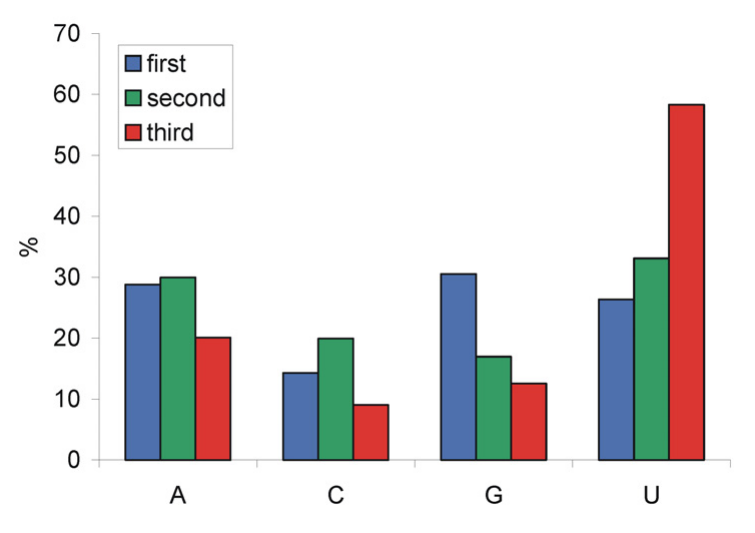
Nucleotide composition of the first, second and third codon positions in the HCoV-NL63 genome.

The bias in the nucleotide count led us to compare the codon usage of HCoV-NL63 with that of human mRNA (Table 1). The codon usage of HCoV-NL63 differs markedly from that of human mRNAs. Third-base choices in the four-codon families (Thr, Pro, Ala, Gly, Val) provide a convenient example of this contrasting codon usage. For instance, the Gly codons in human mRNAs prefer C (34%) over G (25%), A (25%) and U (16%). In contrast, HCoV-NL63 prefers U (83%) over A (7%), C (8%) and G (2%). This result strongly suggests that the codon usage is shaped directly by the unusual nucleotide composition of the viral genome, that is a high U-count and a low G/C-count. All HCoV-NL63 genes, except for the E gene, follow this trend (Table 1). The coronaviral addiction to the U nucleotide is most prominent in the "free" third position of degenerate codons. For the complete genome, the U-count at the third position is up to 58% whereas the A-count is 20%, G-count is 13% and C-count is only 9% (Figure 5). This illustrates that the U-pressure mainly affects the %C and %G.

**Table 1:** Codon usage of HCoV-NL63 compared with that of human genes

Amino acid	Codon	Human^a^	HCoV-NL63	1ab (20190)	S (4071nt)	ORF3 (678nt)	E (234nt)	M (681nt)	N (1134nt)
Arg	CGA	0.62^b^	0.16	0.12	0.22	0.44	***1.28***	0.00	0.26
	CGC	1.07	0.28	0.21	0.37	0.00	0.00	0.88	1.06
	CGG	***1.16***^c^	0.06	0.04	0.15	0.00	0.00	0.00	0.00
	CGU	0.46	***1.57***	***1.49***	***1.40***	***1.77***	0.00	***2.20***	***3.44***
	AGA	***1.17***	0.78	0.76	0.74	0.88	0.00	1.32	1.06
	AGG	***1.17***	0.47	0.49	0.22	0.00	***1.28***	0.00	1.32
Leu	CUA	0.70	0.39	0.31	0.29	2.65	1.28	0.88	0.26
	CUC	1.97	0.36	0.24	0.74	0.88	2.56	0.44	0.26
	CUG	***4.01***	0.21	0.18	0.37	0.44	1.28	0.00	0.00
	CUU	1.30	***2.97***	2.84	2.87	***4.42***	***3.85***	***5.73***	***2.91***
	UUA	0.74	2.68	2.75	2.51	2.65	***3.85***	4.41	0.79
	UUG	1.28	***2.95***	***2.97***	***3.02***	3.54	2.56	2.20	2.38
Ser	UCA	1.20	1.49	1.38	1.92	1.33	0.00	0.44	2.91
	UCC	1.76	0.33	0.30	0.59	0.00	***1.28***	0.00	0.26
	UCG	0.45	0.08	0.07	0.00	0.44	0.00	0.00	0.26
	UCU	1.49	***3.06***	***2.76***	***3.98***	***2.21***	0.00	***3.52***	***5.82***
	AGC	***1.94***	0.34	0.27	0.59	0.00	0.00	0.88	0.79
	AGU	1.21	2.47	2.51	2.36	***2.21***	***1.28***	3.08	2.12
Thr	ACA	1.49	1.72	1.89	1.33	0.88	***1.28***	***2.64***	0.26
	ACC	***1.91***	0.44	0.40	0.52	0.00	***1.28***	0.44	1.06
	ACG	0.62	0.19	0.13	0.37	0.44	0.00	0.88	0.00
	ACU	1.30	***3.58***	***3.28***	***5.53***	***4.87***	***1.28***	1.76	***2.65***
Pro	CCA	1.68	1.03	1.01	1.11	0.44	***2.56***	0.44	1.59
	CCC	**2.00**	0.18	0.15	0.22	0.00	0.00	0.00	0.79
	CCG	0.70	0.10	0.07	0.22	0.00	0.00	0.44	0.00
	CCU	1.74	***2.10***	***1.93***	***1.92***	***3.10***	1.28	***2.20***	***5.29***
Ala	GCA	1.60	1.51	1.66	1.33	0.00	***2.56***	1.32	0.26
	GCC	***2.83***	0.64	0.55	1.11	0.88	0.00	0.44	0.79
	GCG	0.75	0.14	0.13	0.22	0.00	0.00	0.00	0.26
	GCU	1.86	***3.38***	***3.39***	***3.02***	***3.98***	***2.56***	***2.64***	***4.76***
Gly	GGA	1.64	0.46	0.49	0.44	0.00	0.00	0.44	0.26
	GGC	***2.26***	0.48	0.34	1.18	1.33	0.00	0.44	0.00
	GGG	1.65	0.14	0.13	0.07	0.00	0.00	0.44	0.53
	GGU	1.08	***5.19***	***5.56***	***4.35***	***4.42***	***1.28***	***4.41***	***3.44***
Val	GUA	0.71	1.04	1.19	0.59	0.00	1.28	0.88	0.79
	GUC	1.46	0.89	0.77	0.96	2.65	2.56	1.76	0.79
	GUG	***2.86***	0.77	0.68	1.03	0.88	1.28	2.20	0.26
	GUU	1.10	***7.18***	***7.41***	***6.63***	***7.52***	***3.85***	***7.49***	***5.29***
Lys	AAA	2.40	***3.25***	***3.70***	1.33	***2.65***	***2.56***	1.32	3.70
	AAG	***3.22***	2.25	2.29	***1.77***	1.77	0.00	***1.76***	***4.23***
Asn	AAC	***1.92***	1.27	1.05	2.28	0.44	0.00	0.88	2.38
	AAU	1.67	***4.88***	***4.73***	***6.19***	***3.54***	***3.85***	***3.96***	***4.50***
Gln	CAA	1.2	***1.97***	***1.89***	***2.58***	***1.77***	***5.13***	0.44	1.59
	CAG	***3.44***	1.03	0.76	1.69	0.00	0.00	***2.20***	***3.70***
His	CAC	***1.50***	0.39	0.39	0.52	0.44	0.00	0.00	0.27
	CAU	1.07	***1.50***	***1.6***	***1.03***	***0.88***	***1.28***	***2.64***	***1.06***
Glu	GAA	2.89	***2.20***	***2.35***	***1.55***	***3.10***	***1.28***	***1.32***	2.12
	GAG	***4.00***	1.12	1.17	0.66	0.44	0.00	***1.32***	***2.38***
Asp	GAC	***2.55***	1.19	1.2	1.03	***2.21***	1.28	***1.32***	0.79
	GAU	2.2	***4.27***	***4.67***	***3.10***	1.33	***2.56***	0.88	***5.56***
Tyr	UAC	***1.54***	0.89	0.82	1.03	1.77	1.28	1.32	***1.06***
	UAU	1.21	***3.8***	***3.83***	***4.13***	***5.75***	***5.13***	***3.52***	0.79
Cys	UGC	***1.26***	0.30	0.31	0.29	0.44	0.00	0.00	***0.26***
	UGU	1.03	***3.01***	***3.28***	***3.02***	***1.33***	***2.56***	***1.76***	0.00
Phe	UUC	***2.04***	0.68	0.58	0.74	1.77	2.56	0.88	1.06
	UUU	1.72	***5.76***	***6.02***	***4.94***	***7.52***	***8.97***	***5.29***	***2.65***
Ile	AUA	0.73	1.30	1.29	1.62	1.33	2.56	0.88	0.26
	AUC	***2.11***	0.33	0.30	0.44	0.00	0.00	1.32	0.26
	AUU	1.58	***3.76***	***3.77***	***3.68***	***3.98***	***7.69***	***3.08***	***3.17***

^a ^data obtained from GenBank Release 142.0 [28].

^b ^all values represent the percentage of a specified codon.

^c ^the highest value for each codon group is typed bold.

### Identification of the HCoV-NL63 TRS elements

The 5' end of HCoV-NL63 genome RNA contains the L sequence of 72 nucleotides that ends with the L TRS element. This TRS has a high similarity to short sequences that are located in front of each open reading frame (S-ORF3-E-M-N) [22]. We previously identified the L TRS and body TRS of the N gene using a cDNA bank [6], which allowed us to predict the body TRS of the other genes. To confirm these predictions, we amplified and sequenced all sg mRNA fragments with a general L primer and gene-specific 3' primers in an RT-PCR protocol.

**Figure 6:**
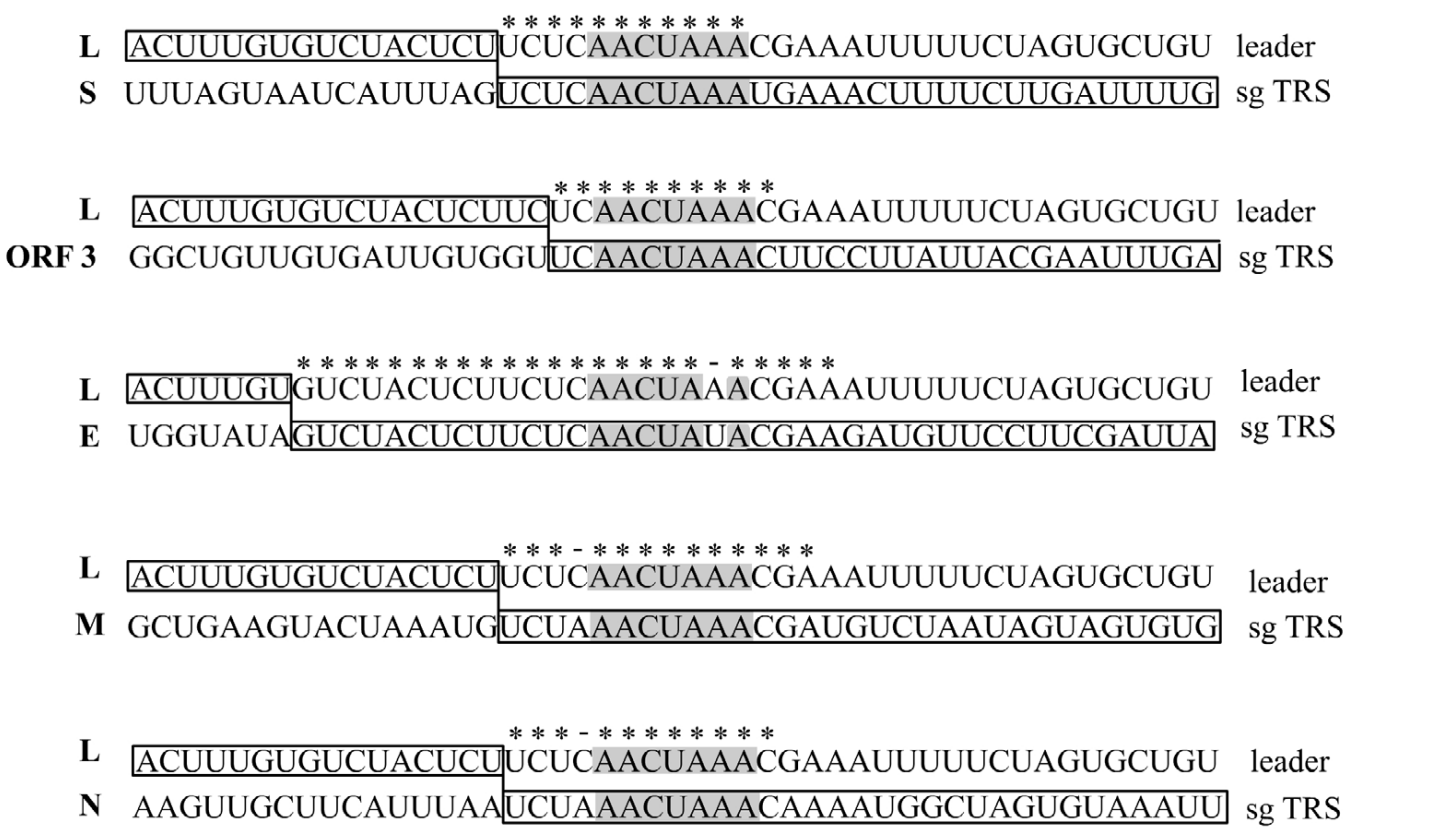
Body-leader junctions of all HCoV-NL63 sg mRNAs. Shown on top is the leader (L) sequence and below the specific sequences upstream of the structural genes. The fusion of 5' L sequences to 3' sg RNA is indicated by the boxes. Sequence homology between the strands near the junction is marked by asterisks, the conserved AACUAAA TRS core is highlighted in gray.

Inspection of sg mRNA junctions indicated that they are indeed composed of the part of the HCoV-NL63 genome that is directly downstream of a particular body TRS, with its 5' end derived from the leader sequence. Apparently, strand transfer occurred on the 5' end of the body TRS, as indicated in Figure 6. The most conserved TRS region was defined by multiple sequence alignment as AACUAAA (gray box). This core sequence is conserved in all sg mRNA, except for the E gene that contains the sub-optimal TRS core AACUAUA (Figure 6). Interestingly, the E gene contains a 13-nucleotide sequence upstream of the core sequence with perfect homology to the L sequence. Perhaps the upstream sequence compensates for the absence of an optimal TRS core during discontinuous (-) strand synthesis. This would suggest that these sequences are copied during (-) strand synthesis, and that the actual strand transfer within the E sequences occurred after copying of the core TRS and the next 13 nucleotides. Evidence for such a "delayed" strand transfer is provided by the junction analysis of the M and N sg mRNAs, which clearly demonstrates that the nucleotides directly upstream of the core TRS are derived from the body TRS element and not from the leader.

### Analysis of the subgenomic mRNAs of HCoV-NL63

**Figure 7:**
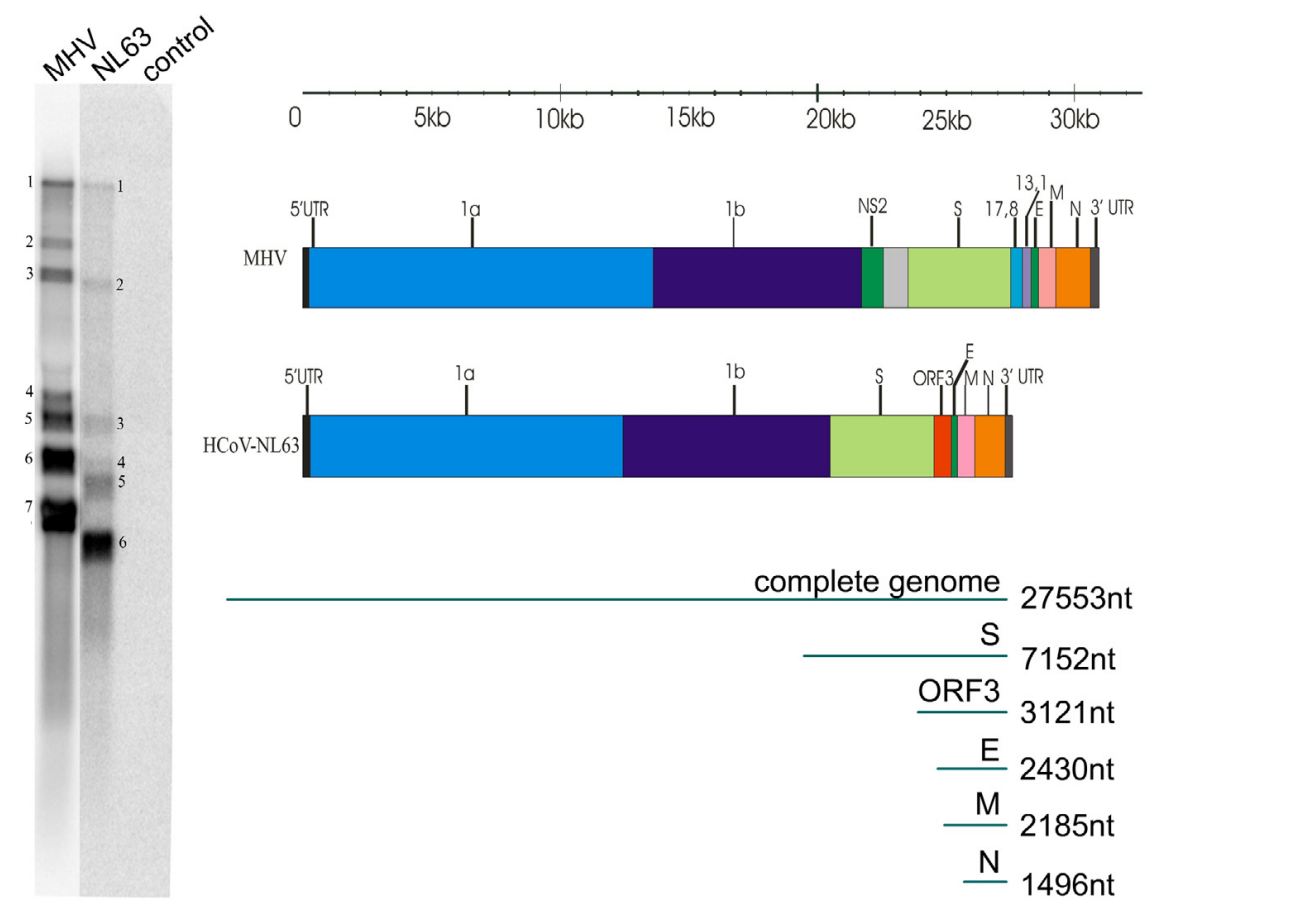
The left panel shows the Northern blot analysis of HCoV-NL63 RNA in infected LLC-MK2 cells. RNA of HCoV-NL63 (NL63 lane) was compared with RNA of MHV strain A59 (MHV lane). Non-infected LLC-MK2 cells are included as a negative control (control lane). MHV RNA bands represent the complete genome (1) and sg mRNAs 2a (2), S (3), 17.8 (4), 13.1 and E (5), M (6), N (7). HCoV-NL63 RNA includes the complete genome (1) and sg mRNAs for S (2), ORF3 (3), E (4), M (5) and N (6). The right panel shows the MHV and HCoV-NL63 genome organization and the HCoV-NL63 sg-mRNAs.

To determine whether the predicted sg mRNAs encoding the S-ORF3-E-M-N proteins are produced in virus-infected cells, we performed Northern blot analysis on total cellular RNA (Figure 7). We used a (-) strand N gene probe that anneals to both genomic RNA and all sg (+) strand mRNAs. We included RNA from MHV-infected cells to obtain discrete size markers. Six distinct mRNAs are produced in HCoV-NL63 infected cells. The sizes of the RNA fragments were estimated and these values nicely fit the size of the genomic RNA and the five predicted sg mRNAs. All HCoV-NL63 ORFs that have the potential to encode viral proteins are indeed transcribed into sg mRNAs (Figure 7).

**Figure 8:**
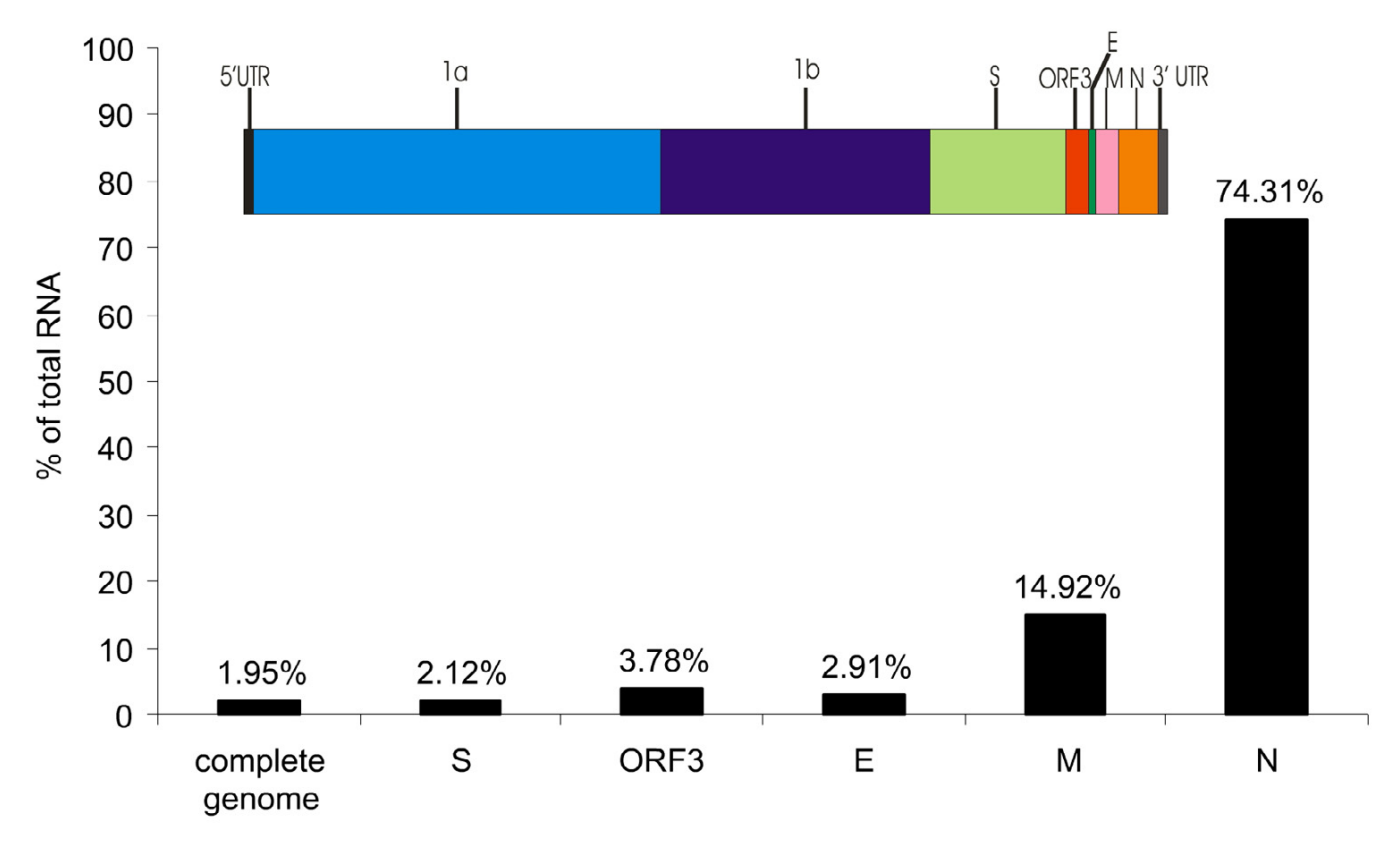
Expression levels of the HCoV-NL63 genomic and sg mRNAs.

To determine the expression level of each subgenomic RNA, we measured the intensity of the signals. When plotted as a function of the genome position (Figure 8), there appears a correlation between the relative distance of a gene to the 3' terminus and its RNA expression level, with the exception of the E gene.

## Discussion

We analyzed the nucleotide composition of the HCoV-NL63 genomic (+) RNA, which was found to exhibit a typical coronavirus pattern with an abundance of U (39 %) and shortage of G (20%) and C (14%). In fact, HCoV-NL63 has the most pronounced nucleotide bias among the coronaviridae.

There is a significant fluctuation in the nucleotide count among the HCoV-NL63 genes. For instance, ORF3 and M appear as extreme U-rich and A-poor islands. It is possible that the unique nucleotide composition of some structural genes reflects their evolutionary origin, perhaps suggesting that some of these functions were acquired recently from another viral or cellular origin by gene transfer. These properties mimic the pathogenicity islands of prokaryotic genomes [23]. Consistent with this gene transfer hypothesis is the observation that there is a lot of variation in the number and identity of the 3' genes among coronaviridae.

Inspection of the nucleotide composition along the genome indicates a bi-phasic pattern. The 5' two-third of the genome encoding the 1ab polyprotein has a stable nucleotide count with the typical U>A>G>C order, but rather striking differences are observed in the 3' one-third of the genome that encodes the structural proteins (Figure 2). Most notably, the C-count increases significantly at the expense of G and U. Grigoriev recently reported the typical nucleotide bias of coronaviral genomes and the switch in nucleotide count at two-thirds of the genome [20]. He performed an analysis based on cumulative GC-skew, and suggested that the drop in GC-ratio is in fact due to a decrease in G-count. However, inspection of the HCoV-NL63 nucleotide composition indicates that the switch is due to a sudden increase in C-count, with a slight drop in G-count. Inspection of other coronaviral genomes confirms that C goes up (with highest significance in group 1 coronaviruses) and G goes down (with highest significance in group 2 coronaviruses) at two-third of the viral genome (results not shown). Grigoriev presented a possible mechanistic explanation. He suggested that the 3'-terminal one-third of the viral genomic (-) strand is more likely to be single stranded because (-) sg mRNA synthesis on the (+) strand template frequently disrupts the protective duplex in that region. This would make this part of the (-) strand genome more vulnerable to C to U transitions, which would eventually lead to a decrease of the G-count on the (+) strand. This scenario explains the G decrease, but obviously is not consistent with the local increase in C-count. We therefore propose an alternative mechanism that is also dictated by the viral transcription strategy. The central 1a/1b portion of the viral (+) strand genome is less likely to be annealed to complementary (-) strand during viral replication because most (-) strand RNAs are sub-genomic, which lack this 1a/1b domain. The 1a/1b portion of the genome thus becomes more vulnerable to C to U deamination, which correlates with the high U-count and the low C-count. Obviously, there may be many other cellular conditions and viral properties like higher amount of secondary structures on the 3' part of the genome that could have shaped the coronavirus genome over an evolutionary timescale, but this scenario explains the switch in nucleotide count at two-thirds of the viral genome.

We show that U-counts reach the highest values and C-counts the lowest values at the third position of the HCoV-NL63 codons (Figure 5). Analysis of the synonymous codon usage indicates that codons with a high U and A content are preferred over C and G rich codons (Table 1). Thus, the peculiar genome composition has a direct effect on the codon usage of HCoV-NL63, and possibly even an indirect effect on the amino acid composition of coronaviral proteins by affecting the non-synonymous codon usage [24-26]. The synonymous codon usage of HCoV-NL63 clearly differs from that in human cells. Thus, the genome may have been shaped by cytosine deamination over an evolutionary timescale, but it is possible that the translational machinery has restricted this genome drift because of the availability of tRNA molecules.

Inspection of the viral genome sequence led us to predict that the 1ab polyprotein is expressed from the genomic RNA and the 3' structural proteins and ORF3 from 5 distinct sg mRNAs. This was confirmed experimentally. We observed that sg mRNAs are more abundant when the corresponding TRS is located closer to the 3' end of the genome. The exception is formed by the E sg mRNA, which is relatively underexpressed. This may correlate with the low expression level of this protein. The general trend of increased gene expression along the genome has been reported previously for other coronaviruses [19]. A possible mechanistic explanation is that the viral polymerase density is reduced along the genome or that the polymerase becomes less susceptible to execute a transfer from body TRS to L TRS during extended (-) strand synthesis. Fine-tuning of the efficiency of the strand-transfer processes may be modulated by many other features, including the local sequence and structure of the core body TRS and its flanking regions. It was reported previously [27] that the core of the L TRS of group 1 coronaviruses is presented in the single stranded loop of a mini-hairpin. We found similar motifs in HCoV-NL63 (results not shown). Although not excessively stable, this structural motifs is predicted to fold as part of the complete leader sequence, and it may participate in the strand transfer process.

The core sequence AACUAAA is conserved in the L TRS and all body TRSs, except for the E gene that has a single mismatch AACUAUA. The presence of a sub-optimal core sequence may in fact explain the lower than expected expression level of this sg mRNA (Figure 8). But there is another striking feature of the E body TRS: it has 13 additional upstream nucleotides in common with the leader TRS. If one assumes that strand transfer does not occur at the core sequence but up to 13 nucleotides further upstream, this sequence homology will result in additional base pairing interactions that may stimulate the strand transfer process. Thus, the extended TRS homology may compensate for the sub-optimal core element. A remarkably similar scenario of sub-optimal core and extended TRS is apparent in the E gene sequence of PEDV (results not shown). A further indication that the viral polymerase frequently copies beyond the core sequence is provided by the actual sequence of the M and N sg mRNAs, which apparently have copied the TRS nucleotide that flanks on the 5' side the core element of body TRS.

## Methods

### Genome Analysis

The nucleotide content of different Coronaviridae family members was assessed using BioEdit software. The nucleotide distribution was determined using a Microsoft Excel datasheet (300 nucleotide (nt) window and 10-nt step). Codon usage was assessed using DNA 2.0 software. Data was processed in Microsoft Excel datasheet and all statistical analysis was performed with SPSS 11.5.0 software. The level of significance of the nucleotide bias was established for 300-nt non-overlapping windows with the non-parametric Mann-Whitney U test for two independent samples. Cumulative GC-skew graphs were generated as described previously [20] with the value in step n defined as the sum of (G-C)/(G+C) from step 0 to n (200-nt sliding window, 10-nt step).

### Viral RNA isolation

HCoV-NL63 RNA was obtained from virus-infected LLC-MK2 cells (2 × 10^7^) after 6 days of culture (virus passage 7). Mouse Hepatitis Virus (MHV) RNA was obtained by infecting 2 × 10^7 ^LR7 cells with MHV strain A59. The medium was removed and the cells were dissolved in 15 ml TRIzol^® ^and RNA was isolated according to the standard TRIzol^® ^procedure. RNA was subsequently precipitated with 0.8 volume of isopropanol, dried and dissolved in 50 μl H_2_O. Integrity of the RNA was analyzed by electrophoresis on a non-denaturating 0.8% agarose gel. RNA was stored at -150°C.

### RT-PCR

The cDNA used for sequencing and probe construction was made by MMLV-RT on viral RNA with 1 μg of random hexamer DNA primers in 10 mM Tris pH 8.3, 50 mM KCl, 0.1% Triton-X100, 6 mM of MgCl_2 _and 50 μM of each dNTPs at 37°C for 1 hour. The single stranded cDNA product was made into double-stranded DNA in a standard PCR reaction with 1.25 U of Taq polymerase (Perkin-Elmer) per reaction with appropriate primers (see below).

### Northern Blot

Gel electrophoresis of viral RNA was performed on a 1% agarose gel with 7% of formaldehyde at 100 Volt in 1×MOPS buffer (40 mM MOPS, 10 mM sodium acetate, pH 7.0). Transfer onto a positively charged nylon membrane (Boehringer Mannheim) was done overnight by means of capillary force. RNA was linked to the membrane in a UV crosslinker (Stratagene). For generation of the HCoV-NL63 probe, the RT-PCR product was further amplified with 5' primer N5PCR1 (CTG TTA CTT TGG CTT TAA AGA ACT TAG G) and 3' primer N3PCR1 (CTC ACT ATC AAA GAA TAA CGC AGC CTG). Similarly, the MHV probe was amplified with 5' primer MHV_UTR-B5' (GAT GAA GTA GAT AAT GTA AGC GT) and 3' primer MHV_UTR-B3' (TGC CAC AAC CTT CTC TAT CTG TTA T). Labeling of the probes was done in a standard PCR reaction with specific 3' primers (N3PCR1 and MHV_UTR-B3') in presence of [α-^32^P]dCTP. Prehybridization and hybridization was done in ULTRAhyb buffer (Ambion) at 50°C for 1 and 12 hours, respectively. The membrane was then washed at room temperature with low-stringency buffer (2×SSC, 0.2% SDS) and at 50°C in high stringency buffer (0.1×SSC, 0.2% SDS). Images were obtained using the STORM 860 phosphorimager (Amersham Biosciences) and data analysis was performed with the ImageQuant software package. The size of sg mRNA fragments of HCoV-NL63 were estimated from their migration on the Northern blot using the sg mRNA of MHV as size marker.

### Sequence analysis of TRS motifs

The L/body TRS junctions were PCR-amplified from an HCoV-NL63 cDNA bank. We performed 35 cycle PCR with the 5' L primer (L5 – TAA AGA ATT TTT CTA TCT ATA GAT AG) and gene specific 3' primers (S gene – SL3' – ACT ACG GTG ATT ACC AAC ATC AAT ATA; ORF3 – 4L3' – CAA GCA ACA CGA CCT CTA GCA GTA AG; E gene – EL3' – TAT TTG CAT ATA ATC TTG GTA AGC; M gene – ML3' – GAC CCA GTC CAC ATT AAA ATT GAC A; N gene – 3-163-F15 – ATT ACC TAG GTA CTG GAC CT). The PCR products were analyzed by electrophoresis on a 0.8% agarose gel and products of discrete size were used for sequencing using the BigDye terminator kit (ABI) and ABI Prism 377 sequencer (Perkin Elmer). Sequence analysis was performed by Sequence Navigator and AutoAssembler 2.1 software.

### Sequences

The complete genome sequence of HCoV-NL63 [6] is deposited in GenBank (accession number: NC_005831). sg mRNA sequences are deposited in GenBank under the accession numbers: AY697419-AY697423. The GenBank accession number of the sequences used in this genome analysis are: MHV (mouse hepatitis virus, strain MHV-A59): NC_001846; HCoV-229E: NC_002645; HCoV-OC43 strain ATCC VR-759: NC_005147; PEDV (porcine epidemic diarrhea virus, strain CV777): AF353511; TGEV (transmissible gastroenteritis virus, strain Purdue): NC_002306; SARS-CoV isolate Tor2: NC_004718; IBV (avian infectious bronchitis virus, strain Beaudette): NC_001451; BCoV (bovine coronavirus, isolate BCoV-ENT): NC_003045.

## Competing interests

The authors declare that they have no competing interests.

## Authors' contributions

KP carried out the viral RNA isolation, RT-PCR, sequencing of sg mRNAs, Northern blot evaluation and all computer analysis done in this study; MFJ carried out the full genome sequencing; all authors participated in writing the manuscript. Lv/dH and BB are the principal investigators
